# Loperamide increases mouse gut transit time in a dose-dependent manner with treatment duration-dependent effects on distinct gut microbial taxa

**DOI:** 10.1017/gmb.2025.5

**Published:** 2025-05-02

**Authors:** Anna Pii Hjørne, Martin Steen Mortensen, Tine Rask Licht, Martin Frederik Laursen

**Affiliations:** National Food Institute, Technical University of Denmark, Kongens Lyngby, Denmark

**Keywords:** gut microbiome, transit time, loperamide

## Abstract

Intestinal transit time has been recognized as an important factor in shaping the gut microbiota, although causality remains to be firmly demonstrated. The aim of this study was to evaluate the effect of different loperamide doses on the mouse intestinal transit time and to investigate the effects of increasing transit time on the gut microbial community. Loperamide significantly increased the transit time in a dose-dependent manner. Additionally, we observed a significant difference between the control group and the loperamide-treated groups in the abundance of the bacterial families *Bacteroidaceae, Erysipelotrichaceae, Porphyromonadaceae*, and *Akkermansiaceae* after 7 days of loperamide treatment, with the bacterial families responding to the increased transit time at different rates. Fermentation of faeces obtained from the same mice, with or without loperamide, demonstrated that the observed effects on gut microbiota *in vivo* were not a result of direct interactions between loperamide and the gut microbiota but rather a consequence of loperamide-induced increased intestinal transit time. In the cecum of the mice, we found higher levels of propionate in the high-dose group compared to the control and low-dose groups. Collectively, our findings establish that an altered transit time is causal to changes in the composition and activity of the microbiome.

## Introduction

Intestinal transit time has been recognized as one of the most important factors to consider when interpreting gut microbiome data, and increasing evidence indicates that gut microbiota signatures relating to specific disease conditions may be a consequence of differences in transit time rather than by the disease *per se* (Procházková et al., 2023). Moreover, intestinal transit time varies considerably between healthy individuals (Procházková et al., 2024), which may partly explain why individuals respond differently to the same dietary interventions. Thus, identifying the complex interactions between transit time and the gut microbiota is essential for understanding the gut microbiota’s role in health and disease.

In human observational studies, differences in colonic transit time (Roager et al., 2016; Procházková et al., 2024), total intestinal transit time (Asnicar et al., 2021; Procházková et al., 2024), and Bristol Stool Scale (Falony et al., 2016; Vandeputte et al., 2016; Müller et al., 2019), used as a proxy for transit time, have all been demonstrated to explain a significant amount of gut microbiota variation. Moreover, a strong association between alpha diversity and intestinal transit time has been found in several studies using different methods to record transit time (Roager et al., 2016; Vandeputte et al., 2016; Müller et al., 2019; Asnicar et al., 2021; Boekhorst et al., 2022; Procházková et al., 2024). Differences are also seen in the metabolic activity of the gut microbiota between individuals with different transit times. Increased levels of proteolytic catabolites are found in individuals with longer colonic transit time (Roager et al., 2016; Procházková et al., 2024), while negative correlations between faecal levels of short-chain fatty acids (SCFAs) and rectosigmoid transit time (Müller et al., 2019) and faecal levels of propionate and colonic transit time (Procházková et al., 2024) have been shown, suggesting that longer transit time changes the fermentative profile of the microbial community.

Although human observational studies are crucial for discovering general patterns and generating hypotheses, controlled experimental setups are necessary to establish causality. Loperamide is the active ingredient in many over-the-counter drugs against diarrhoea, and it increases intestinal transit time by decreasing fluid secretion and peristalsis in the gut (Baker, 2007). In rodent studies, treatment with loperamide hydrochloride is commonly applied to prolong intestinal transit time; however, typically with the purpose of testing the effects of other drugs or food ingredients aimed to relieve constipation rather than to investigate the effect of transit time on the gut microbiota. Although increased transit time has been reported in other animal models, such as the tryptophan hydroxylase 2 knockout mouse (Li et al., 2011) or the inbred mouse strain CFP/Yit (Wagai et al., 2024), important advantages of the loperamide model include its applicability across different rodent strains and the possibility of applying different doses and durations, potentially providing better control of the transit time. In previous mice studies, loperamide has been administered in drinking water (Touw et al., 2017), through oral gavage (Wang et al., 2017a; 2017b; Hayeeawaema et al., 2020; Li et al., 2020; Tang et al., 2022), or by subcutaneous injections (Zhang et al., 2021; Li et al., 2022), with doses ranging between 5–10 mg/kg body weight. While some differences in the gut microbiome and metabolome have already been reported in studies of loperamide-treated mice, these measures have usually not been the primary focus of the studies (Wang et al., 2017a; 2017b; Hayeeawaema et al., 2020; Li et al., 2020; Zhang et al., 2021; Li et al., 2022; Tang et al., 2022), and these studies did not rule out the option that observed effects on the gut microbiota might be due to direct effects of loperamide. In addition, no studies have so far investigated the dose–response relationship of loperamide on gut microbial changes in mice. Therefore, the aim of this study was to investigate and validate the effect of loperamide-induced increased intestinal transit time on the mice gut microbiota and its metabolites and examine whether potential dose effects of the drug on transit time would also manifest in any changes to the gut microbiome.

## Materials and methods

### Animals and experimental design

The study was conducted at the Technical University of Denmark with ethical approval from the Danish Animal Experiment Inspectorate (permit number 2020-15-0201-00484). The in-house Animal Welfare Committee for Animal Care and Use oversaw the experiment, and the experiment was conducted in accordance with the ARRIVE guidelines (Percie du Sert et al., 2020).

Twenty-four female Mouse Pathogen Free C57BL/6 mice, 7–8 weeks of age, were purchased from Taconic Biosciences (Ejby, Denmark). The software G*power 3 (Faul et al., 2007) was used to estimate the necessary sample size for the experiment, with intestinal transit time as the main outcome. With a power of 0.80 and an alpha level of 0.05, the minimum group size for detecting differences in transit time between the four groups was estimated to be 6 animals per group. Upon arrival, the mice were allowed to acclimatize to the facilities for 11 days before starting the experiment. The mice were kept at a 12-hour light cycle in a constant environment with a relative humidity of 55 ± 5%, a temperature of 22 ± 1 °C, and an air change of 50 times per hour. The animals had ad libitum access to a regular chow diet (Altromin 1314, Brogaarden ApS, Denmark) and drinking water, also during transit time observations. The animals were single-housed during the entire study to limit stress-related effects on the transit time measurements, as expected to occur if the animals were moved from their cage mates during transit time observations.

**Figure 1.**
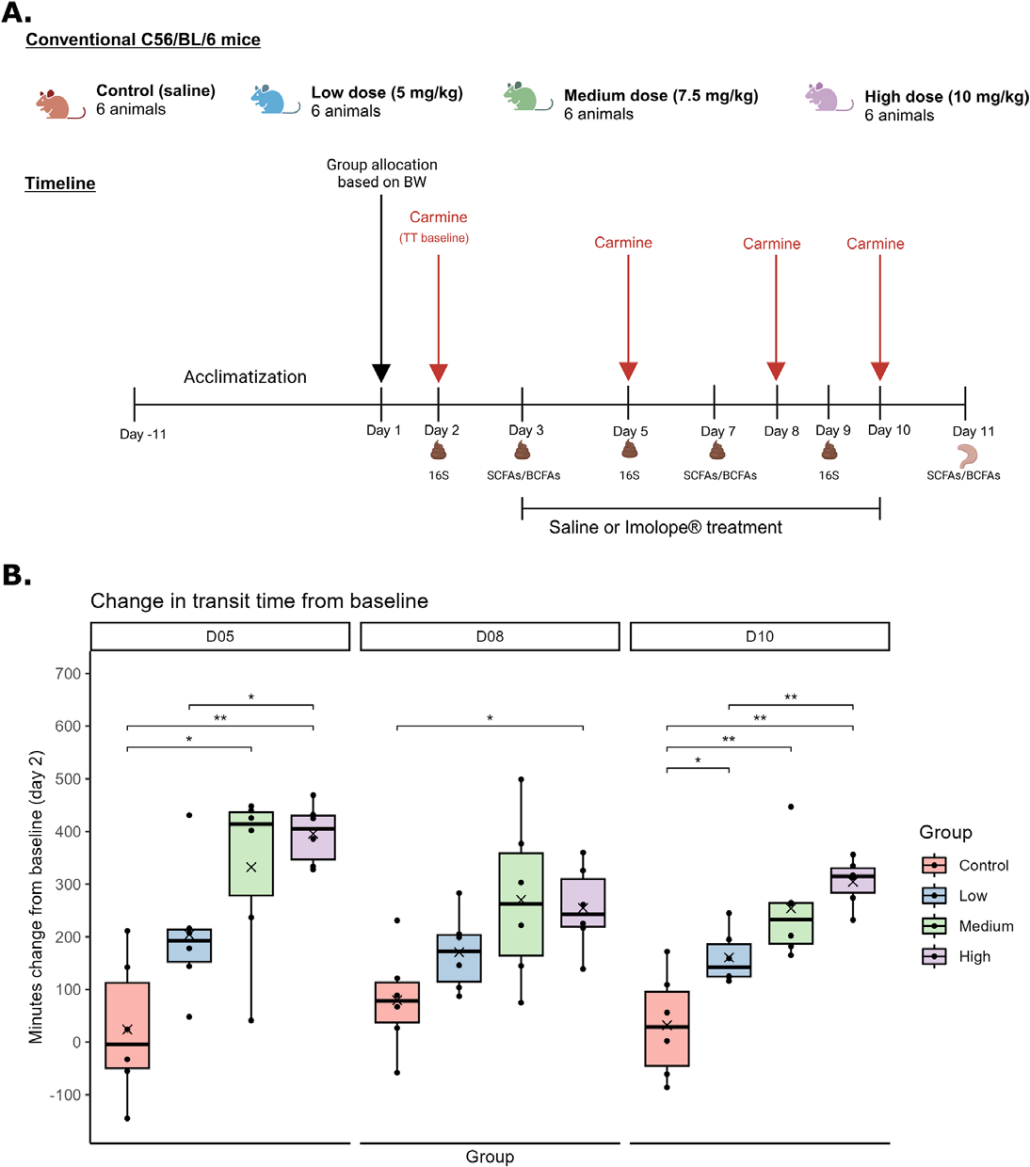
Overview of the study design (A) Minutes change in transit time between baseline observation (Day 2) and observations on Day 5, Day 8, and Day 10 for all groups (B) Differences between the groups were tested with a two-way ANOVA, followed by unpaired t-tests with FDR adjustment. *p < 0.05, **p < 0.01. Figure 1A was created in BioRender. Hjørne, A. (2023) BioRender.com/c29x987.

On Day 1 of the experiment, the animals were allocated into four groups: control (saline), low loperamide dose (5 mg/kg), medium loperamide dose (7.5 mg/kg), and high loperamide dose (10 mg/kg). The animals were allocated to the four groups based on body weight, ensuring a similar average body weight in all groups. Loperamide/saline was administered to the animals through oral gavage for 1 week, starting from Day 3 of the study. Total intestinal time was measured at 4 different time points during the experiment, as described in the next section. Fresh faecal samples were collected on the morning of all days of the experiment, although samples could not be obtained from all animals on all days during the period of loperamide treatment due to constipation. The samples were kept on ice during collection and immediately transferred to −20 °C after collection. Bodyweight and food intake were monitored daily during the experiment. The experiment was terminated in the morning on Day 11, where all animals were anesthetised with hypnorm/midazolam (0.1 ml/10 g SC) and euthanized by cervical dislocation after collection of heart blood. The experimental design is illustrated in Figure 1A.

### Oral administration and transit time estimation

In a pilot experiment (data not shown), we found that it was not possible to dissolve loperamide hydrochloride purchased from Sigma-Aldrich in saline or Tween, and the pure compound could not be used for oral administration. Instead, we used pulverised Imolope® tablets from Orifarm Generics, containing 2 mg of loperamide hydrochloride per tablet. The tablets were pulverized and dissolved in sterile saline in three different suspensions (0.05%, 0.075%, and 0.1% loperamide) to ensure similar dosing volumes for the three treatment groups. Dosing volume was calculated each day based on the body weight from the day before. Control animals received a volume of sterile saline corresponding to the volume of loperamide solution administered to animals of similar body weight, and dosing volumes ranged from 180–230 μL.

A solution of 6% carmine red (Sigma-Aldrich, C1022) and 0.05% methylcellulose (Sigma-Aldrich, M0262) was prepared the day before transit time measurements. The effect of the carmine red solution on the gut microbiome has already been tested in a previous animal study (Dey et al., 2015), which found no significant effects of the solution on the gut microbiome. On the morning of transit time measurements, the animals were moved to a clean cage with white absorbent paper covering the cage floor. All animals were administered 150 μL of the carmine red solution through oral gavage. On the days of co-administration of the loperamide and carmine solutions, loperamide was administered first, and faecal pellets were collected before administration. The time of carmine red gavage was recorded for each animal, and the animals were observed every 10–15 mins until the first red faecal pellet appeared. The transit time was calculated as the time between oral administration and the appearance of the first red pellet. The researcher observing the animals during transit time measurements was blinded to the treatment status of the animals.

### In vitro test of direct drug effects on the gut microbiome

We performed an anaerobic batch fermentation experiment to test for any direct effects of the Imolope® solution on the gut microbiome. The experiment included three groups: control (saline), low loperamide (0.3 mg/l), and high loperamide (3 mg/l). The chosen loperamide concentrations were based on a paper from 1979 demonstrating that 70% of radiolabelled loperamide administered to rats was taken up in the liver or tissue (Miyazaki et al., 1979), leaving 30% for transit through the colon before being excreted in the faeces. Thus, the 3 mg/ml corresponds to 30% of the 10 mg/kg body weight dosed to the high-dose mouse group in this study. The Imolope® solution was prepared as described for the animals above. Saline or Imolope® solution was added to 15 ml Falcon tubes containing pre-reduced-modified Gifu Anaerobic Medium (mGAM broth, Shimadzu, 05433-GBM-0100) with a pH of 7.3. A total volume of 5 ml was used for the fermentation, and all conditions were set up in triplicates. A faecal slurry was prepared by pooling faeces collected from all control animals on Day 4 of the experiment. Faecal pellets were homogenised in pre-reduced PBS (250 μL per pellet) inside an anaerobic chamber, and 50 μL of the faecal slurry was inoculated into the media with saline/Imolope®. The tubes were allowed to ferment while shaking for 72 hours in total, and 500 μL samples were collected every 24 hours. Immediately after collection, the samples were centrifuged at 10,000 g for 10 mins at 4 °C, the supernatant was removed, and the pellets were kept at -20 °C until DNA extraction. An overview of the fermentation experiment is provided in Supplementary Figure 10.

### DNA extraction

The Qiagen Powersoil DNeasy kit (Qiagen) was used to extract DNA from mouse faecal samples (12–270 mg) and from the samples collected during the *in vitro* experiment. Before DNA extraction, faecal samples were suspended in 4x volumes of sterile Milli-Q, followed by centrifugation at 16,000 g for 10 mins. The supernatant was aspirated, and the pellet was used for DNA extraction. A blank DNA extraction control was included, and the manufacturer’s instructions were followed with a few adjustments, described in the following. Samples were homogenised by 10 mins of bead beating on a Retsch MM300 mixer mill (30/s) using the glass beads provided in the kit. In the first centrifugation step, samples were centrifuged at 16,000 g for 3 mins. In all other centrifugation steps, samples were centrifuged at 16,000 g for 1 min. DNA concentrations were measured using the Qubit 2.0 dsDNA High Sensitivity (Qubit HS) kit (Qiagen) and adjusted to 5 ng/μL for amplicon library preparation.

### 16S rRNA gene amplicon sequencing

PCR amplification and Ion Torrent GSS5 sequencing of the V3 region of the 16S rRNA were performed as previously described (Laursen et al., 2017). Briefly, a Mastermix of 10.4 μL PCR grade water, 4 μL HF-buffer, 0.4 μL dNTP (10 mM), 2 μL reverse primer (PBR 5′-trP1-adapter-ATTACCGCGGCTGCTGG-3′, 10 pmol/ μL), and 0.2 μL Phusion High-Fidelity Polymerase (Fisher Scientific, F-553 L) per sample was prepared. PCR was performed in a 20 μL reaction with 1 μL template DNA (5 ng/μL), 2 μL forward primer (PBU 5′-A-adapter-TCAG-barcode-CCTACGGGAGGCAGCAG-3′, 10 pmol/ μL), and 17 μL Mastermix. The forward and reverse primers were obtained from TAG Copenhagen A/S. Both primers were linked to adaptors necessary for ion torrent sequencing (A-adapter and trP1-adapter), while the forward primer also contained a barcode of 10 bp, unique for each sample. Primers were modified from Milani et al. (2013). A Mock Community (ZymoBIOMICS™ Microbial Community DNA Standard, D6505) was included as a positive control, while PCR-grade water and a blank DNA extraction control were included as negative controls. The PCR was run on the following 45 mins program: (i) 30s denaturation at 98 °C, (ii) 24 cycles of 98 °C for 15 s, and 72 °C for 30s, (iii) 5 mins extension at 72 °C, and (iv) cooling to 4 °C. Randomly selected PCR products were quality-checked on a pre-made 2% agarose E-Gel Power Snap with SYBR-safe gel stain (Thermo Fisher Scientific). The PCR products were purified with HighPrep PCR Magnetic Beads (MAGBIO, AC-60005) using a 96-well magnet stand (MAGBIO, MyMag 96), following the manufacturer’s instructions. Final DNA concentrations were measured using the Qubit 2.0 dsDNA High Sensitivity (Qubit HS) kit (Qiagen), and samples were pooled in equimolar concentrations before Ion Torrent sequencing. Sequencing was performed on a 318-chip using the Ion OneTouch™ 200 bp Template Kit v2 DL.

### Quantitative PCR measurement of total bacterial load

Quantitative PCR (qPCR) was performed to estimate the total faecal bacterial load, using the universal primers PBU (5′-CCTACGGGAGGCAGCAG-3′) and PBR (5′-ATTACCGCGGCTGCTGG-3′). A Mastermix of 6 μL PCR grade water, 1 μL forward primer (PBU, 4 μM), 1 μL reverse primer (PBR, 4 μM), and 10 μL SYBR Green Master Mix (Applied Biosystems, A25742) per sample was prepared. The PCR was performed in a 20 μL reaction with 18 μL Mastermix and 2 μL template DNA (5 ng/μL). A standard curve was generated from a 10-fold serial dilution (10^3^–10^6^ 16S rRNA copies/μL) of known concentrations of DNA from *E. coli* type strain (DSM18039). PCR-grade water was included as a negative control, and all reactions were performed in triplicates. The plate was run on the QuantStudio5™ Real-Time PCR system (Applied Biosystems) with the following program: (i) preincubation at 95 °C for 5 mins, (ii) 40 cycles of 95 °C for 10s, 60 °C for 15 s, and 72 °C for 45 s, (iii) a melting curve analysis of 95 °C for 5 s, 68 °C for 1 mins, and 98 °C for 15 s, and (iv) cooling to 4 °C. Data was analysed with the Design & Analysis software (v2.6.0, Applied Biosystems) and Excel. The qPCR data was used to estimate the absolute abundances of the microbial taxa by multiplying with the relative abundances obtained from the 16S rRNA gene amplicon sequencing.

### Bioinformatic analysis

Raw 16S rRNA amplicon data was processed with an in-house pipeline (Mortensen, 2023). Briefly, demultiplexing was performed with cutadapt (v. 4.1) (Martin, 2011), denoising was performed with DADA2 (v. 1.22) (Callahan et al., 2016), and amplicon sequence variants (ASVs) were classified using the rdp_train_set_18 (Cole et al., 2014). Further processing and analysis of the data were done in R (v. 4.3.1).

### Short-chain and branched-chain fatty acid extraction and analysis

Faecal and caecal water for SCFA and BCFA analysis was prepared by diluting the content in 4x volumes of sterile MiliQ, followed by 1–2 mins of vortexing. Samples were centrifuged at 16.000 g for 10 mins at 4 °C. The supernatant was transferred into 0.22 μm centrifuge filters (Costar Spin-X, centrifuge tube cellulose acetate filters) and filtered by centrifugation at 15.000 g for 10 mins at 4 °C.

Sample analysis was carried out by MS-Omics (Vedbæk, Denmark) as follows. Samples were acidified using hydrochloride acid, and deuterium-labelled internal standards were added. All samples were analysed in a randomized order. Analysis was performed using a high-polarity column (Zebron™ ZB-FFAP, GC Cap. Column 30 m × 0.25 mm × 0.25 μm) installed in a GC (7890B, Agilent) coupled with a time-of-flight MS (Pegasus® BT, LECO). The system was controlled by ChromaTOF® (LECO). Raw data was converted to netCDF format using Chemstation (Agilent), before the data was imported and processed in Matlab R2021b (Mathworks, Inc.) using the PARADISe software described by Johnsen and coworkers (Johnsen et al., 2017).

### Statistics

All statistical analyses were performed in R (v. 4.3.1). All p-values were adjusted for multiple comparisons using the False Discovery Rate (FDR) method. P-values below 0.05 after adjusting for multiple comparisons were considered significant. Appropriate parametric or non-parametric tests were applied for all analyses, depending on normality and variance tests. The tests applied for the specific comparisons are indicated in the figure legends. In all box plots, the border of the boxes indicates the interquartile range (IQR), horizontal lines represent the median, and the whiskers extend from the 25th and 75th percentiles to the furthest outlier within 1.5 times the IQR. The dots represent individual data points, and group means are visualised with a cross.

For beta diversity analysis, we first applied one-way ANOVA to identify possible group differences in beta dispersion, which can affect the interpretation of the following PERMANOVA (Warton et al., 2012). A pairwise PERMANOVA within the Ecole package (Smith, 2021) was performed for beta diversity analysis to test for differences between the animal groups on Day 9 and between the *in vitro* conditions at the three different time points.

The DAtest package (Russel et al., 2018) was first used for the differential abundance analysis to find the best method for analysing the animal data. However, since the DAtest cannot be trusted when there is a separation associated with the predictor (in this case, treatment groups) (Russel et al., 2018), the method identified in the DAtest was only used to screen the data for relevant taxa, i.e., taxa that appeared to differ in relative abundance between the treatment groups on Day 9 of the study. The relative distribution of the identified taxa was transformed into absolute abundances by multiplying with the bacterial load. Differences between the treatment groups in absolute abundances of the identified taxa were tested with appropriate statistical tests, as indicated in the figure legends. Moreover, the trajectories of the taxa were evaluated for all groups, and correlation analysis was used to evaluate associations between transit time and absolute abundances (transit time on Day 5 versus abundance on Day 5 and average transit time on Day 8/10 versus abundance on Day 9). The trajectories of relevant taxa were also evaluated for the different *in vitro* groups through appropriate statistical tests, as indicated in the figure legends.

## Results

### Loperamide treatment induces dose-dependent increases in the gastrointestinal transit time of mice

For an overview of the study design, the reader is referred to Figure 1A. Transit time observations were carried out for all animals four times during the study period (Day 2, Day 5, Day 8, and Day 10). The changes in transit time between the baseline observation (Day 2) and the observations during the treatment period (Day 5, Day 8, and Day 10) were calculated for each animal (Figure 1B). On Day 5, the transit time increased in all treatment groups compared to the control group; however, this increase was only significant for the medium-dose and the high-dose groups. The increase for the high-dose group was significantly higher than for the low-dose group. On Day 8, similar tendencies were observed, although only the difference between the control and high-dose groups was significant following adjustment for multiple comparisons. On Day 10 of the experiment, all treatment groups differed significantly from the control group, and the high-dose and the low-dose groups differed significantly. Together, these results indicate that loperamide increases intestinal transit time dose-dependently in mice. Within the treatment groups, the effect of loperamide on transit time did not accumulate during the treatment period (Day 5 to Day 10). The only observed difference within the groups during the period of loperamide treatment was a decrease between Day 5 and Day 10 in the high-dose group. (Supplementary Figure 1). No significant differences in body weight or food intake were observed between the groups during the study (Supplementary Figure 2).

### Loperamide treatment affects the gut microbiota composition but not the alpha diversity in mice

16S rRNA amplicon sequencing was performed on faecal DNA collected before (Day 2) and during (Day 5 and Day 9) the treatment period. When comparing the alpha diversity (Observed ASVs and Shannon index) on Day 9, no significant differences were observed between the groups (Supplementary Figure 3). Alpha diversity decreased in all groups during the study, but this was not statistically significant following correction for multiple comparisons (Supplementary Figure 3).

**Figure 2.**
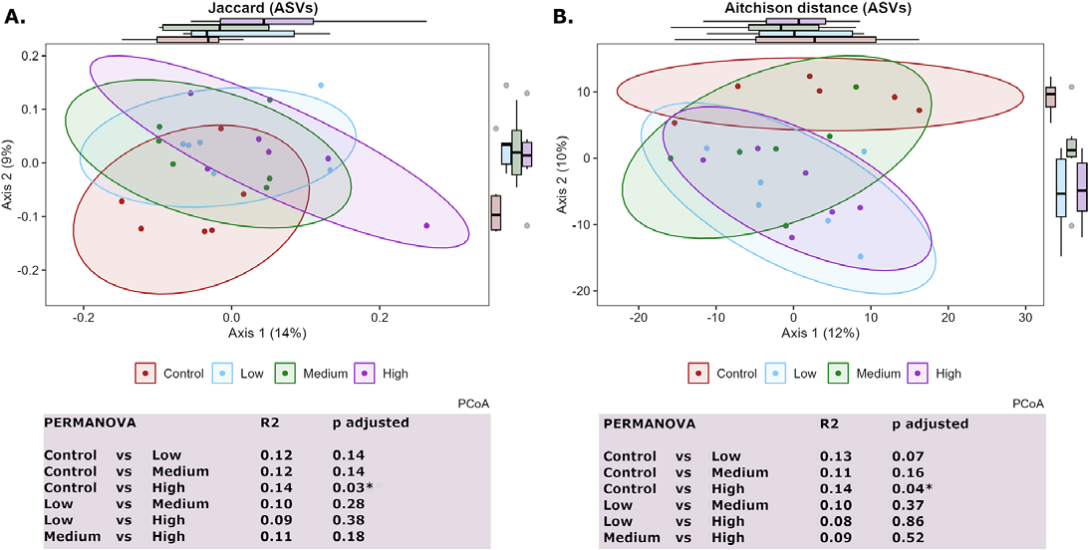
PCOA plots illustrating the Jaccard distance (A) and the Aitchison distance (B) between the faecal microbiotas of all animals on Day 9 (ASV level). Dots represent individual samples, whereas ellipses represent the 90% confidence intervals around the group centroids. Marginal boxplots are included to illustrate the data distribution along the two axes. Differences between the groups in the faecal microbiome composition were tested with pairwise PERMANOVAs with FDR adjustment for multiple comparisons. The R^2^ values indicate the proportion of variance explained by the model.

The beta diversity of the microbial communities originating from the different groups on Day 9 was analysed using Jaccard distances (Jaccard, 1912) (Figure 2A) and robust Aitchison distances (Martino et al., 2019) (Figure 2B). There was a significant difference between the control group and the high-dose group at the ASV level. The low-dose and medium-dose groups followed the same trend, but the differences were not significant after correction for multiple comparisons.

### The abundance of specific bacterial taxa is increased after loperamide treatment in mice

Bacterial load (16S rRNA gene copies/g faeces) did not differ significantly between the groups at any specific time points, but when comparing the bacterial load over time within each group, a significant increase was observed for the high-dose group between Day 5 and Day 9 (Supplementary Figure 4).

**Figure 3.**
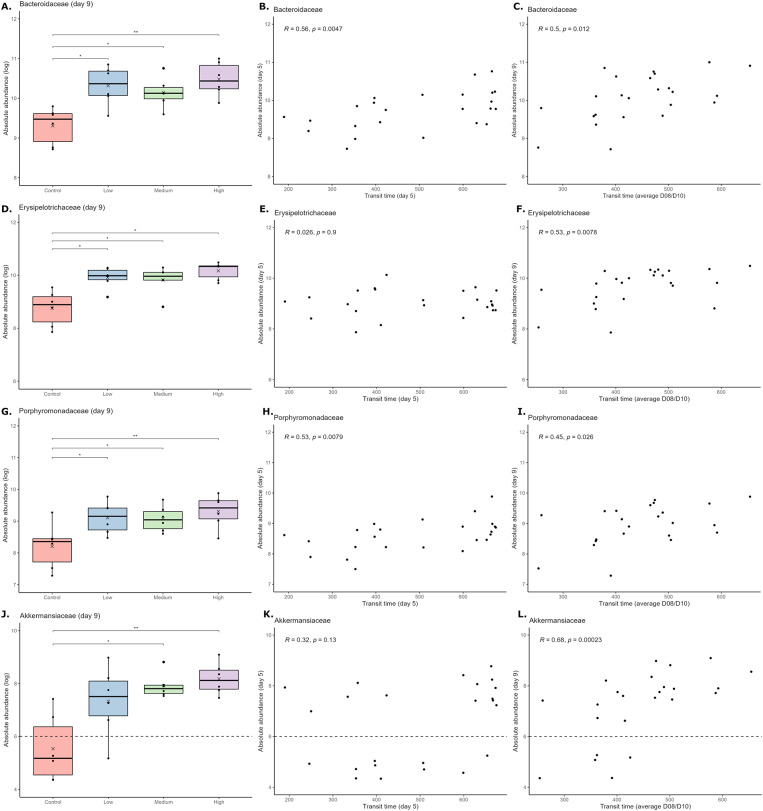
The absolute abundance of *Bacteroidaceae* (A), *Erysipelotrichaceae* (D), *Porphyromonadaceae* (G), and *Akkermansiaceae* (J) in the different groups on Day 9. For *Bacteroidaceae* (A) and *Porphyromonadaceae* (G), differences between the groups were tested through one-way ANOVAs, followed by unpaired t-tests with FDR adjustment for multiple comparisons. For *Akkermansiaceae* (J) and *Erysipelotrichaceae* (D), differences between the groups were tested through Kruskal–Wallis tests, followed by Dunn’s tests with FDR adjustment for multiple comparisons. *p < 0.05, **p < 0.01. Figures B, E, H, and K illustrate the association between the absolute abundances of the taxa and the transit time on Day 5. Figures C, F, I, and L illustrate the association between the absolute abundances of the taxa on Day 9 and the average transit time on Day 8/Day 10. Spearman’s correlation analyses were used to examine the relationship between the variables. For *Akkermansiaceae* (J, K, L), all samples with 0 counts were set to 0.5 counts (LOD) before calculating the abundance and log-transforming the data. The dotted lines indicate the detection limit, meaning that in all samples below this line, no *Akkermansiaceae* was detected.

On Day 9, the absolute abundances of *Bacteroidaceae* (Figure 3A), *Erysipelotrichaceae* (Figure 3D), and *Porphyromonadaceae* (Figure 3G) were significantly higher in the low, medium, and high loperamide treatment groups than in the control group. For *Akkermansiaceae*, the difference was observed for the medium and high treatment groups (Figure 3J). For all these families, similar patterns were observed using the relative abundances (Supplementary Figure 5). No significant differences in absolute and relative abundances of these taxa were observed at baseline (Day 2) (Supplementary Figure 6). Within the *Bacteroidaceae* family, the two genera *Phocaeicola* and *Bacteroides* were found to drive the increase of the family (Supplementary Figure 7A–F). In the *Erysipelotrichaceae* family, *Longibaculum* was the only genus identified to follow a similar pattern (Supplementary Figure 7G–I), although the differences observed for the genus were smaller than observed for the family, indicating that unidentified genera of the *Erysipelotrichaceae* family also contributed to the observed differences. Within the *Porphyromonadaceae* family, only one genus, *Parabacteroides*, was identified, and for *Akkermansiaceae*, only one species, *Akkermansia muciniphila*, was identified. Thus, *Parabacteroides* and *A. muciniphila* naturally followed the exact same pattern as their respective families (Figure 3G–I + Figure 3J–L).

**Figure 4.**
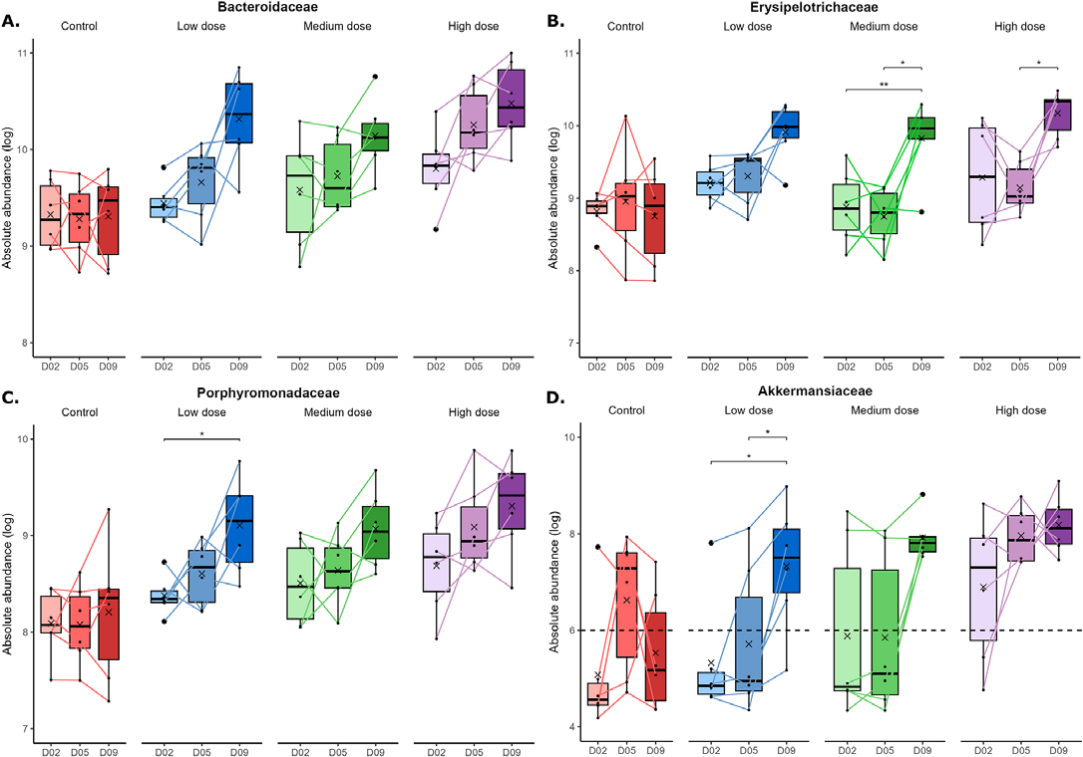
The absolute abundance of *Bacteroidaceae* (A), *Erysipelotrichaceae* (B), *Porphyromonadaceae* (C), and *Akkermansiaceae* (D) on Day 2, Day 5, and Day 9 for all groups. For *Bacteriodaceae*, *Erysipelotrichaceae*, and *Porphyromonadaceae*, differences between the days were tested through two-way ANOVAs, followed by paired t-tests with FDR adjustment for multiple comparisons. For *Akkermansiaceae*, differences between the days were tested through a Friedman test, followed by paired Wilcoxon tests with FDR adjustment for multiple comparisons. *p < 0.05, **p < 0.01. For *Akkermansiaceae* (D), all samples with 0 counts were set to 0.5 counts (LOD) before calculating the abundance and log transforming the data. The dotted line indicates the detection limit, meaning that in all samples below this line, no *Akkermansiaceae* was detected.

We observed significant positive correlations between transit time and absolute abundance of *Bacteroidaceae* (Figure 3B + 3C) and *Porphyromonadaceae* (Figure 3H + 3I) on both Day 5 and Day 8/Day 10. In contrast, these associations were only present on Day 8/Day 10 for *Erysipelotrichaceae* (Figure 3F) and *Akkermansiaceae* (Figure 3L). The absolute abundance of *Bacteroidaceae* (Figure 4A) and *Porphyromonadaceae* (Figure 4C) tended to increase in a stepwise manner over time, whereas the increases in abundance for *Erysipelotrichaceae* (Figure 4B) and *Akkermansiaceae* (Figure 4D) were generally not observed before Day 9 of the experiment.

### Fecal bacterial taxa affected in mice are not directly affected by loperamide in vitro

Since the gut microbiota changes induced by loperamide treatment *in vivo* may reflect direct interactions between loperamide or other components of the Imolope® tablets and the gut microbiota, we tested the potential direct effects of Imolope® tablets on the faecal microbiota community composition using faecal samples obtained from the same mice.

In the *in vitro* setup, the number of observed OTUs was significantly lower in the Imolope® conditions compared to the saline condition after 48 hours of fermentation, and the Shannon diversity was significantly lower in the Imolope® conditions after 72 hours of fermentation (Supplementary Figure 11). When analysing the distance matrices (Supplementary Figure 11), no significant separations between the three conditions were observed at any time point. When evaluating the absolute abundance of *Bacteroidaceae*, *Erysipelotrichaceae*, *Porphyromonadaceae*, and *Akkermansiaceae* over time (Supplementary Figure 13), no significant differences were observed between the time points in any of the test conditions.

### High-dose loperamide treatment increases caecal propionate levels but does not affect the levels of other SCFAs or BCFAs in mice

**Figure 5.**
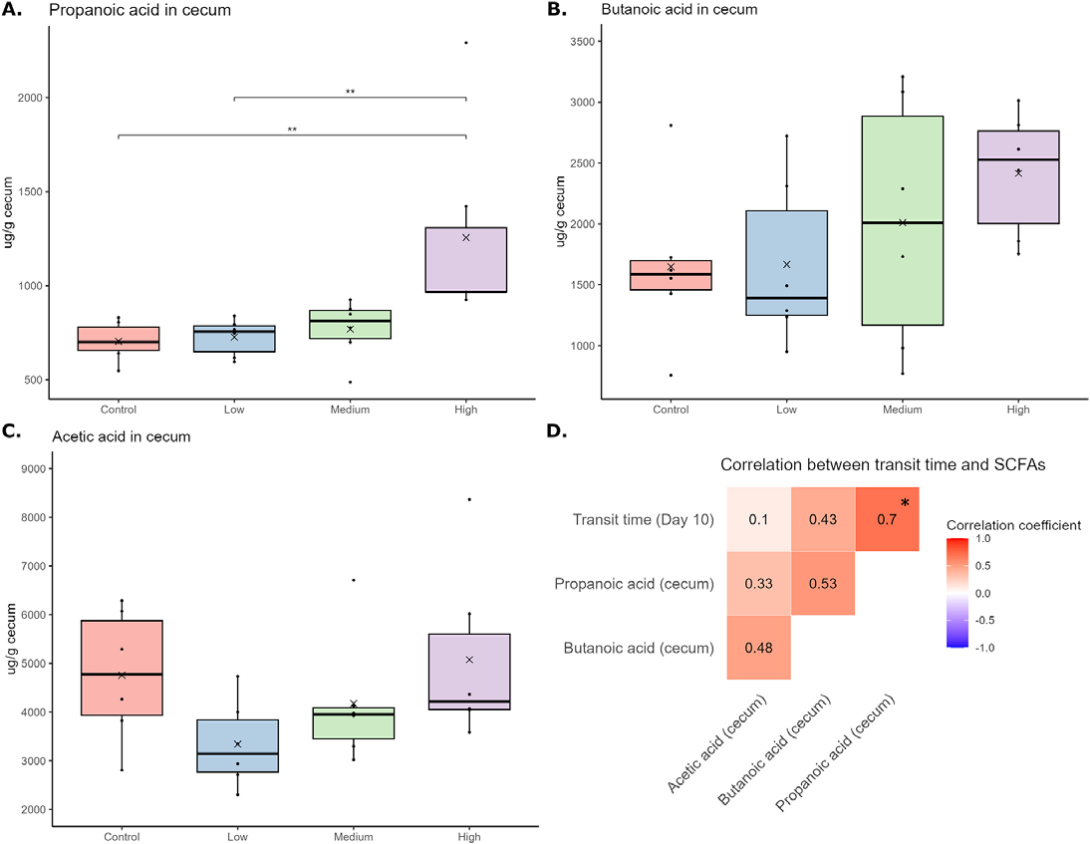
The caecal level of the SCFAs propionate (A), butyrate (B), and acetate (C) in the different groups at the end of the experiment. For propionate (A) and butyrate (B), differences between the groups were tested with one-way ANOVAs, followed by unpaired t-tests with FDR adjustment for multiple comparisons. Two outliers with high propionate levels were identified and removed from the high-dose group before the statistical analysis. For acetate (C), differences between the groups were tested with a Kruskal–Wallis test (not significant). In Figure D, the correlations between transit time for all animals on Day 10 and the caecal level of the SCFAs are illustrated. Spearman’s correlation analyses with FDR adjustment for multiple comparisons were used to examine the relationship between the variables. *p < 0.05, **p < 0.01.

Changes in the levels of SCFAs (butyrate, acetate, and propionate) and BCFAs (isobutyrate and isovalerate) between faeces collected on Day 3 (before treatment was initiated) and Day 7 (during the treatment period) were calculated for each animal (Supplementary Figure 8). No significant differences in the faecal levels of the SCFAs or BCFAs were observed between the groups for any of the metabolites. However, caecal propionate was significantly increased in the high-dose group as compared to the control and low-dose groups (Figure 5A), and a significant positive correlation was observed between caecal propionate levels and transit time on Day 10 (Figure 5D). There was a tendency for increased caecal butyrate in the medium- and high-dose groups (Figure 5B, ANOVA p-value = 0.3) and a tendency for a positive correlation between butyrate and transit time (Figure 5D, adjusted Spearman p-value = 0.06). No differences in caecal acetate (Figure 5C) or caecal BCFAs (Supplementary Figure 9) were observed between the groups.

## Discussion

We demonstrated a dose-dependent effect of loperamide on increased intestinal transit time in mice. It should be noted that the applied carmine red method of recording transit time is not very precise and that transit time exhibits high inter- and intra-individual variation (Procházková et al., 2023, 2024). Factors known to affect transit time in humans include physical activity and stress (Procházková et al., 2023), and even small disturbances in the animal’s environment or differences in their activity level can have affected the transit time observations. Therefore, caution should be taken when interpreting the exact transit time values.

We did not observe any significant differences in alpha diversity between the groups, which contrasts with observations from humans, where increased transit time consistently has been linked to increased alpha diversity (Roager et al., 2016; Vandeputte et al., 2016; Müller et al., 2019; Asnicar et al., 2021; Boekhorst et al., 2022; Procházková et al., 2024). Previous mouse studies applying loperamide-induced constipation have reported both unchanged (Touw et al., 2017; Wang et al., 2017b), increased (Li et al., 2020), and decreased (Zhang et al., 2021; Tang et al., 2022) alpha diversity. In a specific inbred mouse model with increased transit time, a lower alpha diversity has also been reported (Wagai et al., 2024). Potential explanations for the inconsistency between the mice studies are differences in mouse strains, diet compositions, or housing conditions, such as the single housing used in our experiment, which has prevented the animals from exchanging microbes with each other. With respect to the inconsistency between human and mice studies, it is important to consider that human intestinal transit times of several days are normal (Procházková et al., 2023, 2024), whereas transit times for mice with a loperamide-induced increase do not exceed 24 hours. Since human diets are also more complex and diverse than experimental animal diets, longer transit times in humans might support a higher availability of microbiota-accessible nutrients and the growth of more different species.

Loperamide treatment led to an increase in relative and absolute abundances of the bacterial families *Bacteroidaceae*, *Porphyromonadaceae*, *Erysipelotrichaceae*, and *Akkermansiaceae* in the faeces of mice from all treatment groups. In other loperamide studies in mice, increased abundances of *Bacteroidaceae* (Touw et al., 2017; Hayeeawaema et al., 2020)*, Porphyromonadaceae* (Touw et al., 2017; Wang et al., 2017b; Zhang et al., 2021), and *Erysipelotrichaceae* (Zhang et al., 2021) have also been reported, although reports on a decreased (Zhang et al., 2021) or unchanged (Wang et al., 2017b) abundance of *Bacteroidaceae* genera also exists. In faecal cultures exposed to the Imolope® solution *in vitro*, we found no significant increase in the absolute abundance of the identified taxes over time. Moreover, our results align with reports from other models or human cohorts. In a study on an inbred mouse strain with spontaneously increased intestinal transit time, an increased abundance of *Erysipelotrichaceae* was observed (Wagai et al., 2024), similar to our findings. Increasing transit time *in vitro* led to an increased abundance of *Bacteroides, Parabacteroides*, and *Akkermansia* (Minnebo et al., 2023), also in line with our observations. In human cohorts, an increase of *Bacteroides* (Asnicar et al., 2021) and *Akkermansia* (Falony et al., 2016; Vandeputte et al., 2016; Asnicar et al., 2021; Procházková et al., 2024) with increased transit time has been reported. Together, this strongly suggests that differences in intestinal transit time, and not direct effects of loperamide or the Imolope® additives on the gut microbiota, drove the reported differences in this study.

For *Bacteroidaceae* and *Porphyromonadaceae*, we found a significant positive correlation between transit time and abundance already on Day 5, whereas a similar response to increased transit time was delayed for *Erysipelotrichaceae* and *Akkermansiaceae.* A potential explanation for the differences in response time could be different growth rates of the given taxa. *Bacteroides* spp. are, for example, recognised for their relatively rapid growth rate, whereas *Akkermansia* has a slow *in silico* predicted growth rate (Minnebo et al., 2023). Alternatively, the slow responders might be dependent on the faster responders, for example, through cross-feeding of complex carbohydrates. It is well described that *Bacteroides* spp. can utilize a wide variety of carbon sources (Wexler, 2007; Flint et al., 2012), which provides nutrients for other bacteria in cross-feeding interactions (Flint et al., 2012; Sinha et al., 2024). The ability to feed on more complex carbon sources may also explain why *Bacteroides* spp. can thrive at longer transit times where simple carbon sources are likely to become scarce. Similarly, *Erysipelotrichaceae* has been found to increase in faeces from mice supplemented with the (nonfermentable) fibre hydroxypropyl methylcellulose (Cox et al., 2013) and in faeces from mice supplemented with both short-chain and long-chain inulin (Li et al., 2020a), while a positive correlation between *Porphyromonadaceae* abundance and faecal SCFAs has been found in humans (Kelder et al., 2014). For *Akkermansiaceae*, the ability to grow on mucins (Derrien et al., 2004) may give the taxa an advantage when other microbes compete for the luminal content during a long intestinal passage.

Our observation of increased caecal propionate levels in the high-dose loperamide group is another finding where our study contrasts observations from humans, linking increased transit times to increased levels of proteolytic catabolites in faeces and urine (Roager et al., 2016; Procházková et al., 2024) and decreased levels of SCFAs in faeces (Müller et al., 2019; Procházková et al., 2024). Similarly, decreased faecal SCFAs have been reported in other animal studies with loperamide-induced constipation (Zhang et al., 2021; Tang et al., 2022). However, in functional analysis of shotgun metagenomic sequencing data from human faeces, a higher rate of pyruvate-to-propionate fermentation was found in individuals with long transit times (Asnicar et al., 2021). It has been suggested that the low faecal levels of SCFAs observed with longer intestinal transit time can be explained by more time for absorption of the SCFAs, since increased propionate and acetate levels were found in an *in vitro* system with long transit time (Minnebo et al., 2023). This may explain why we observed increased propionate levels in the caecum but not in the faeces of the mice. Moreover, the dietary fibre content of the chow diet used in our study may be too high to allow for the depletion of fermentable carbohydrates, resulting in the shift to proteolytic fermentation reported in humans (Roager et al., 2016).

In conclusion, our findings establish that an altered transit time in itself causes changes in the composition and activity of the mouse microbiome, and we ruled out the possibility that the observed effects might be due to direct interactions between loperamide and the gut microbiota. The longitudinal sampling in this study revealed that the response of different taxes to increased transit time happened at different rates. Furthermore, we demonstrated that the dose of loperamide was not only important for transit time but also for the effects on the gut microbiota. The loperamide-induced increased intestinal transit time model presented here can be used conceptually to understand how transit time may explain differences in gut microbiota responses to specific dietary interventions.

## Supporting information

Hjørne et al. supplementary material Hjørne et al. supplementary material

## Data Availability

Sequencing data have been deposited at the NCBI Sequence Read Archive under the Bioproject ID PRJNA1152344 (https://www.ncbi.nlm.nih.gov/bioproject/1152344), and all R scripts used for the data analysis are available on data.dtu.dk (DOI: 10.11583/DTU.28741808).

## Supplementary material

The supplementary material for this article can be found at http://doi.org/10.1017/gmb.2025.5.
