# Supplementary material for: Loperamide increases mouse gut transit time in a dose-dependent manner with treatment duration-dependent effects on distinct gut microbial taxa
Source: Gut Microbiome (Camb). 2025 May 2;6:e7. doi: 10.1017/gmb.2025.5 (PMC12056420; doi:10.1017/gmb.2025.5)
Supplement: Hjørne et al. supplementary material [file S2632289725000052sup001.docx]

**Supplementary figures**

**
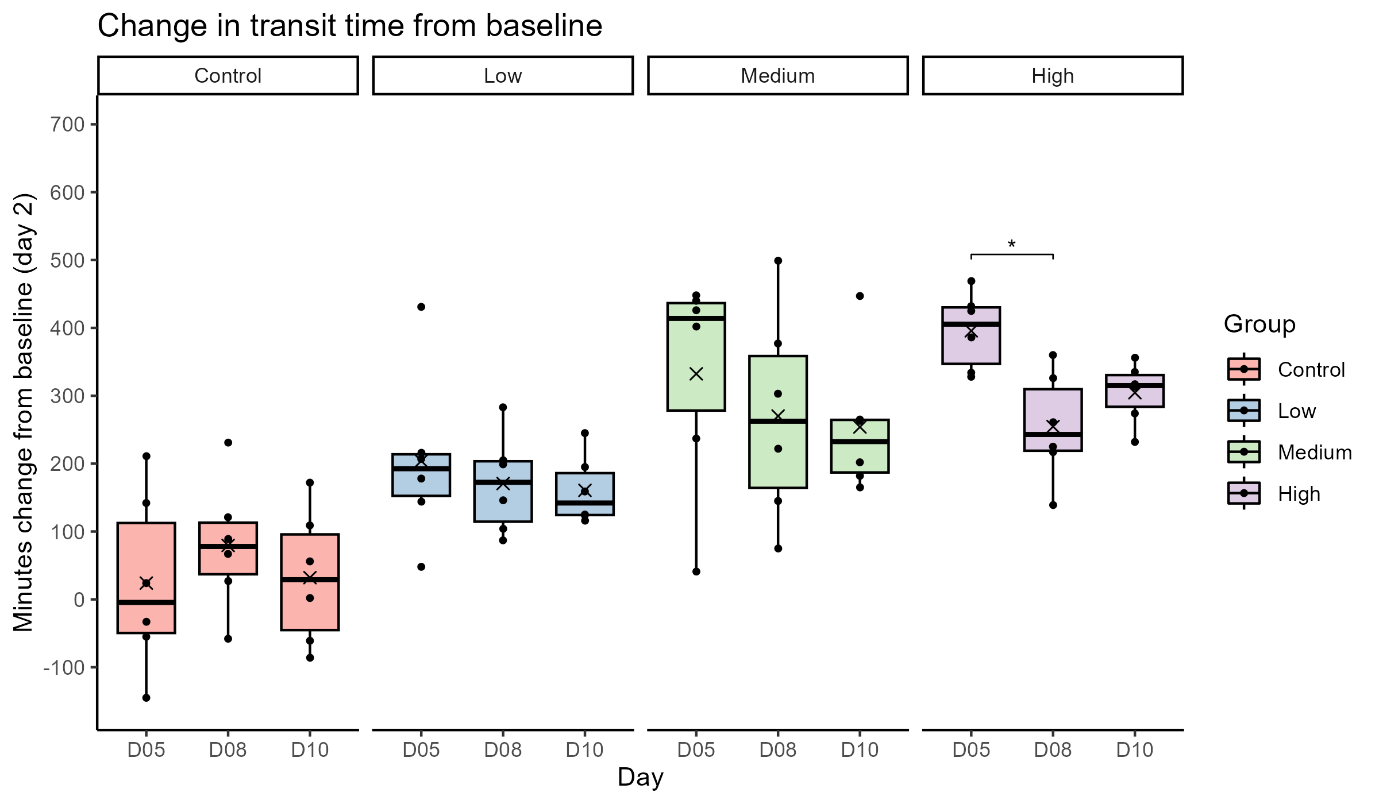
**

*Supplementary figure 1. The change in transit time between baseline (Day 2) and the different days of treatment (Day 5, Day 8, and Day 10) for all treatment groups. Differences between the days were tested with a two-way ANOVA, followed by paired t-tests with FDR adjustment. *p<0.05.*


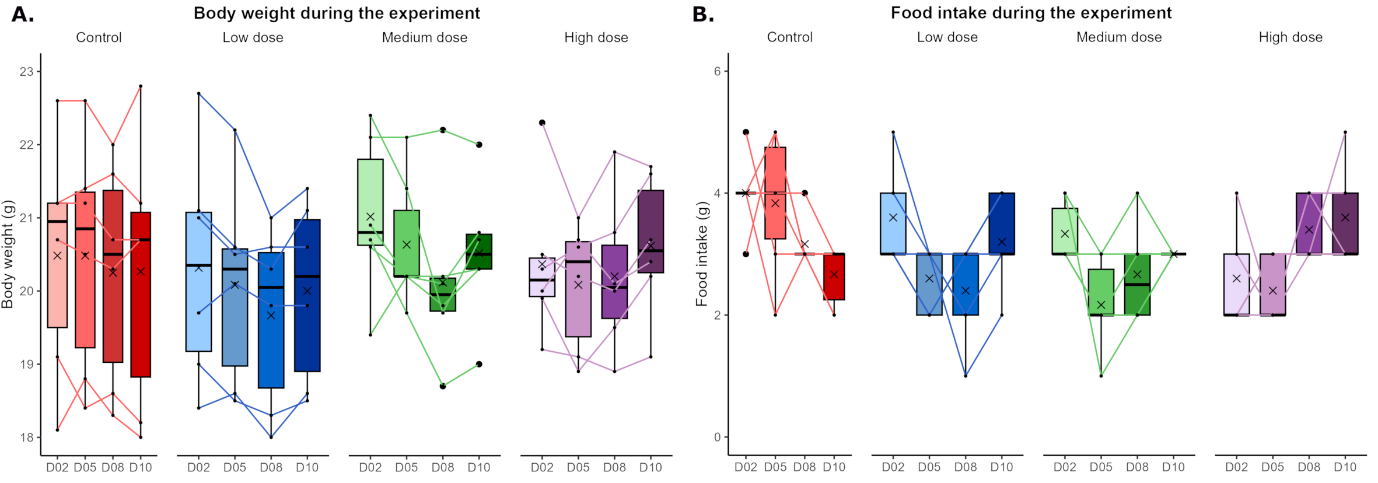


*Supplementary figure 2. The body weight (A) and food intake (B) in all groups on Day 2, Day 5, Day 8, and Day 10. Differences between the different days were tested with a Friedman’s test (non-significant).*


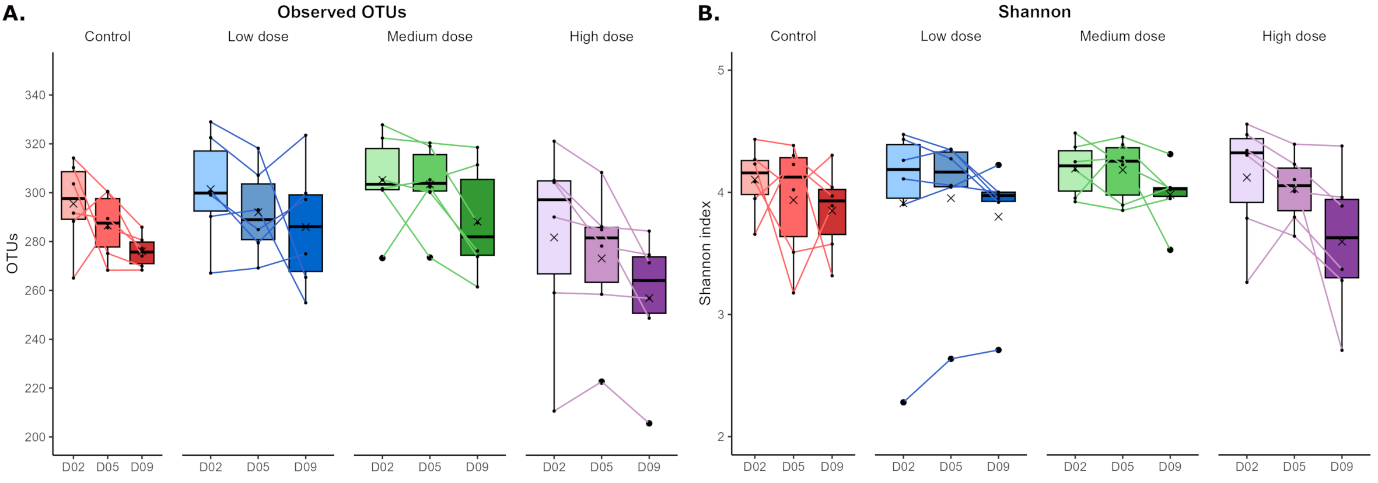


*Supplementary figure 3. The number of observed OTUs (A) and the Shannon index (B) in the different groups on Day 2, Day 5, and Day 9. For observed OTUs (A), differences were tested with a two-way ANOVA and paired t-tests for post hoc analysis. For the Shannon index (B), differences were tested with Friedman’s test (non-significant).*


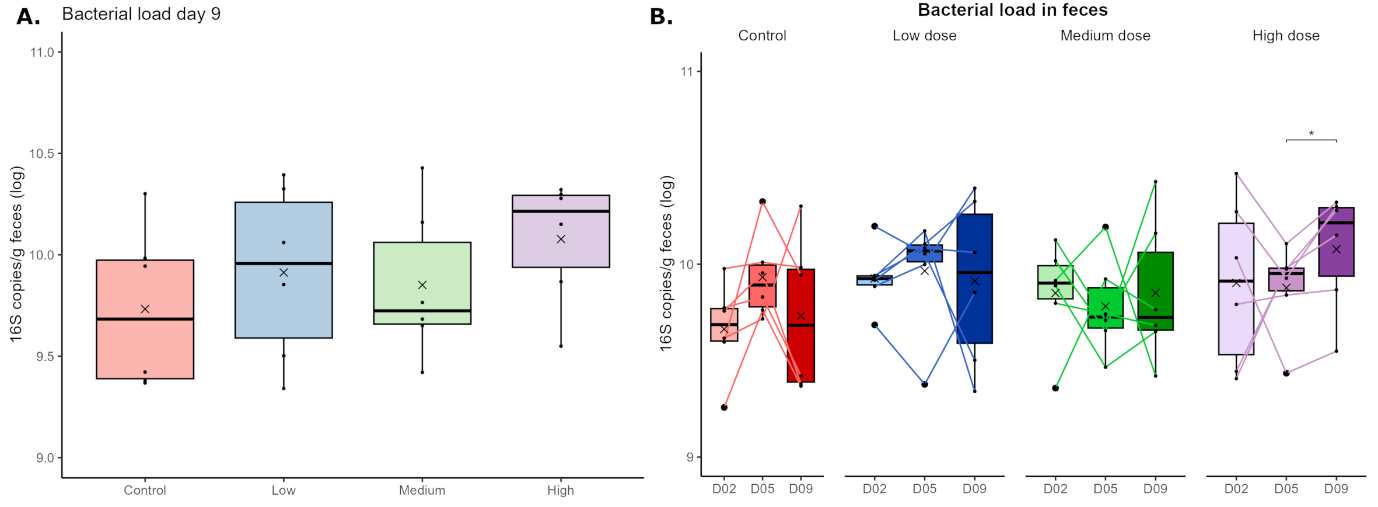


*Supplementary figure 4. Bacterial load (16S rRNA gene copies/g feces, log-transformed) for all groups on Day 9 (A) and for all groups on Day 2, Day 5, and Day 9 (B). For Figure A, differences between the groups were tested with a Kruskal-Wallis test (non-significant). For Figure B, differences between the days were tested with a two-way ANOVA, followed by paired t-tests with FDR adjustment for multiple comparisons. All y-axes are on a logarithmic scale. *p<0.05.*


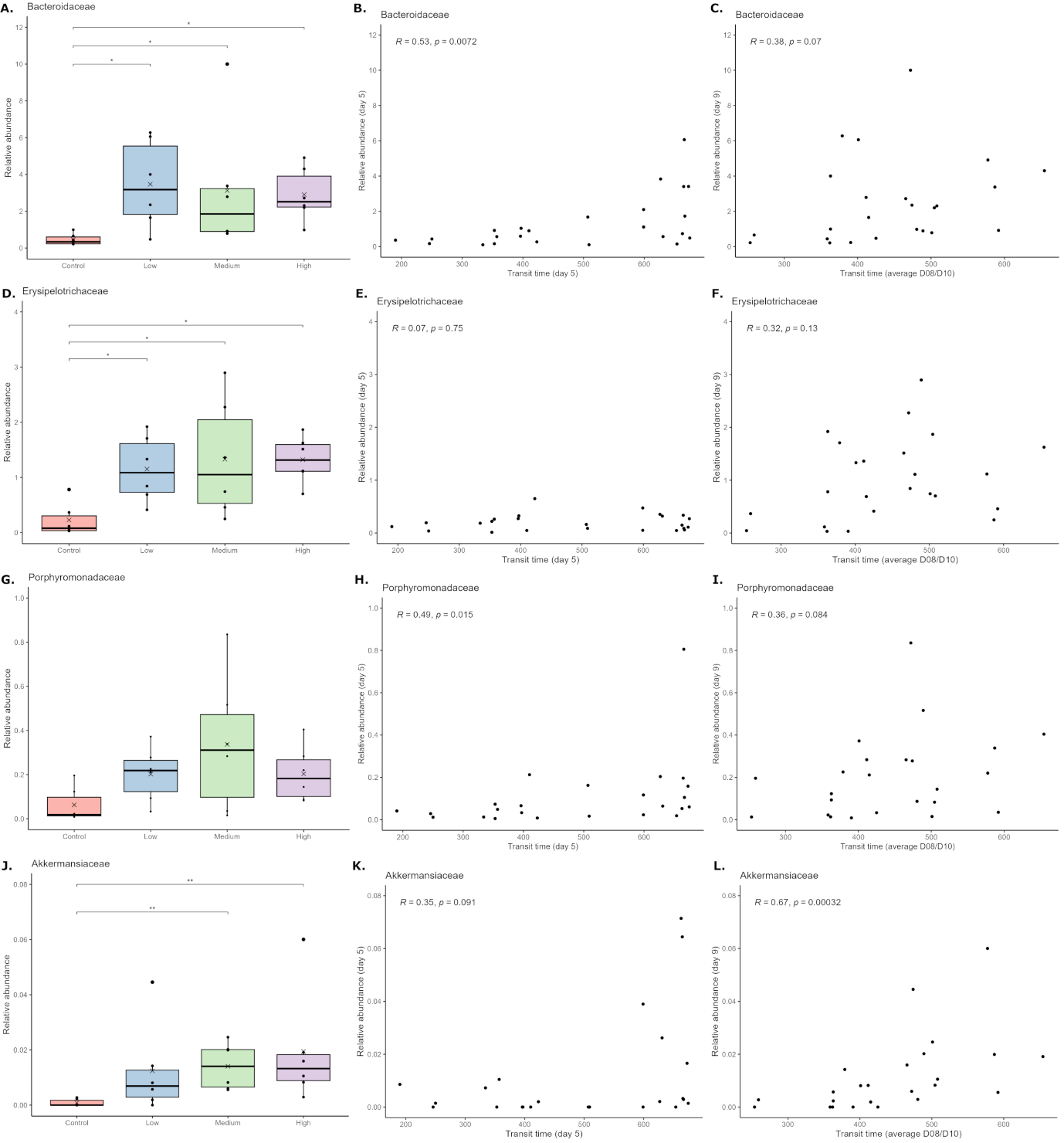


*Supplementary figure 5. The relative fecal abundances of Bacteriodaceae (A), Erysipelotrichaceae (D), Porphyromonadaceae (G), and Akkermansiaceae (J) for all groups on Day 9. Differences between the groups were tested through Kruskal-Wallis tests, followed by Dunn’s tests with FDR adjustment for multiple comparisons. *p<0.05, **p<0.01.* *Figures B, E, H, and K illustrate the association between the relative abundances of the taxa and the transit time on Day 5. Figures C, F, I, and L illustrate the association between the relative abundances of the taxa on Day 9 and the average transit time on Day 8/10. Spearman’s correlation analyses were used to examine the relationship between the variables.*

*
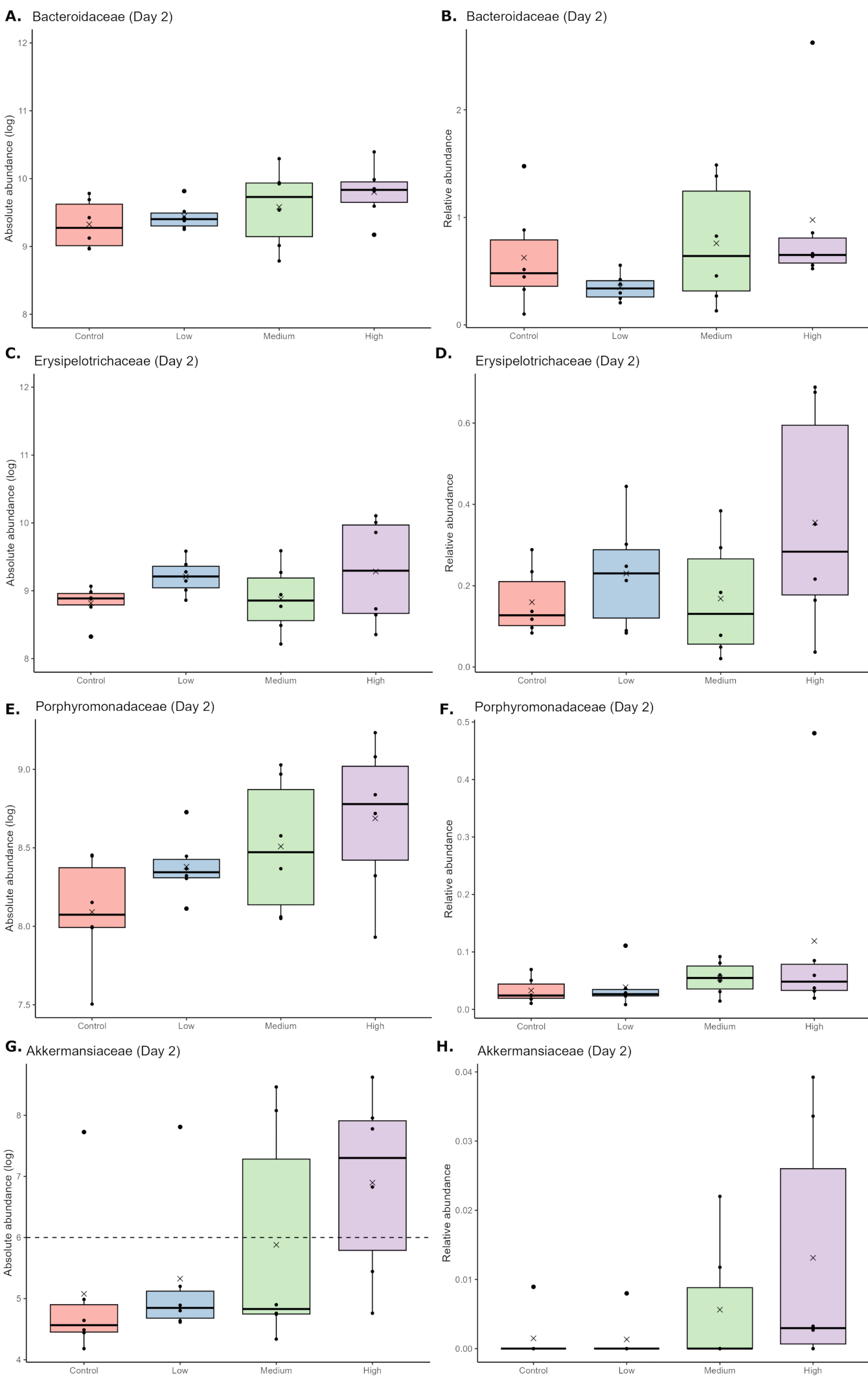
*

*Supplementary figure 6. The absolute and relative fecal abundances of Bacteriodaceae (A-B), Erysipelotrichaceae (C-D), Porphyromonadaceae (E-F), and Akkermansiaceae (G-H) for all groups on Day 2. For the absolute abundance of Bacteriodaceae (A) and Porphyromonadaceae (E), differences between the groups were tested with one-way ANOVAs, followed by unpaired t-tests with FDR adjustment for multiple comparisons. For all other analyses, differences between the groups were tested through Kruskal-Wallis tests, followed by Dunn’s tests with FDR adjustment for multiple comparisons.*

**
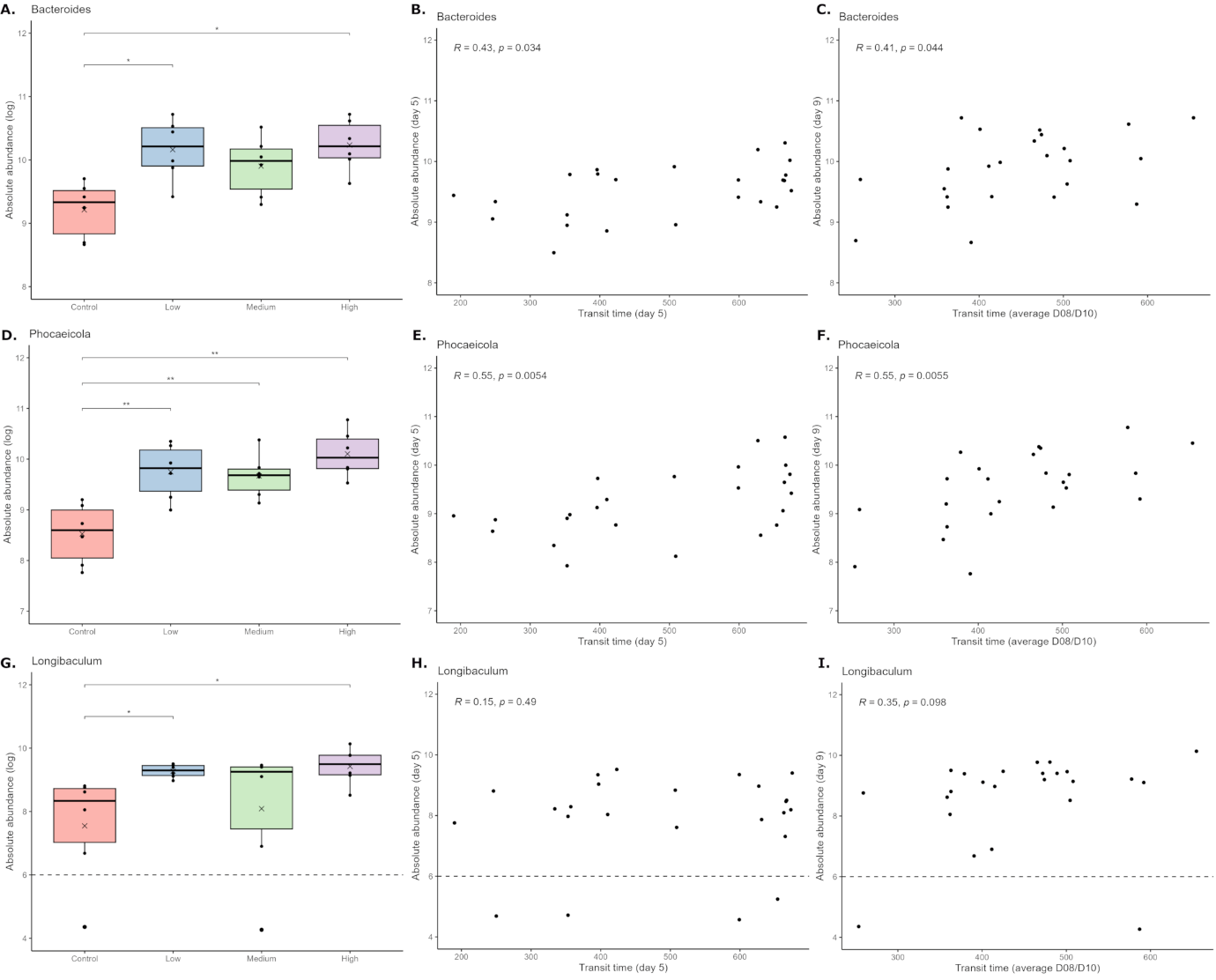
**

*Supplementary figure 7. The absolute fecal abundance of Bacteroides (A), Phocaeicola (B), and Longibaculum (C) for all groups on Day 9. For Bacteroides (A) and Phocaeicola (B), differences between the groups were tested through a one-way ANOVA, followed by unpaired t-tests with FDR adjustment for multiple comparisons. For Longibaculum differences between the groups were tested through a Kruskal-Wallis test, followed by Dunn’s test with FDR adjustment for multiple comparisons. *p<0.05, **p<0.01. Figures B, E, H, and K illustrate the association between the absolute abundances of the taxa and the transit time on Day 5. Figures C, F, I, and L illustrate the association between the absolute abundances of the taxa on Day 9 and the average transit time on Day 8/10. Spearman’s correlation analyses were used to examine the relationship between the variables. All y-axes are on a logarithmic scale. For Longibaculum (G, H, I), all samples with 0 counts were set to 0.5 counts (LOD) before calculating the abundance and log-transforming. The dotted lines indicate the detection limit, meaning that in all samples below this line, no Longibaculum was detected.*

**
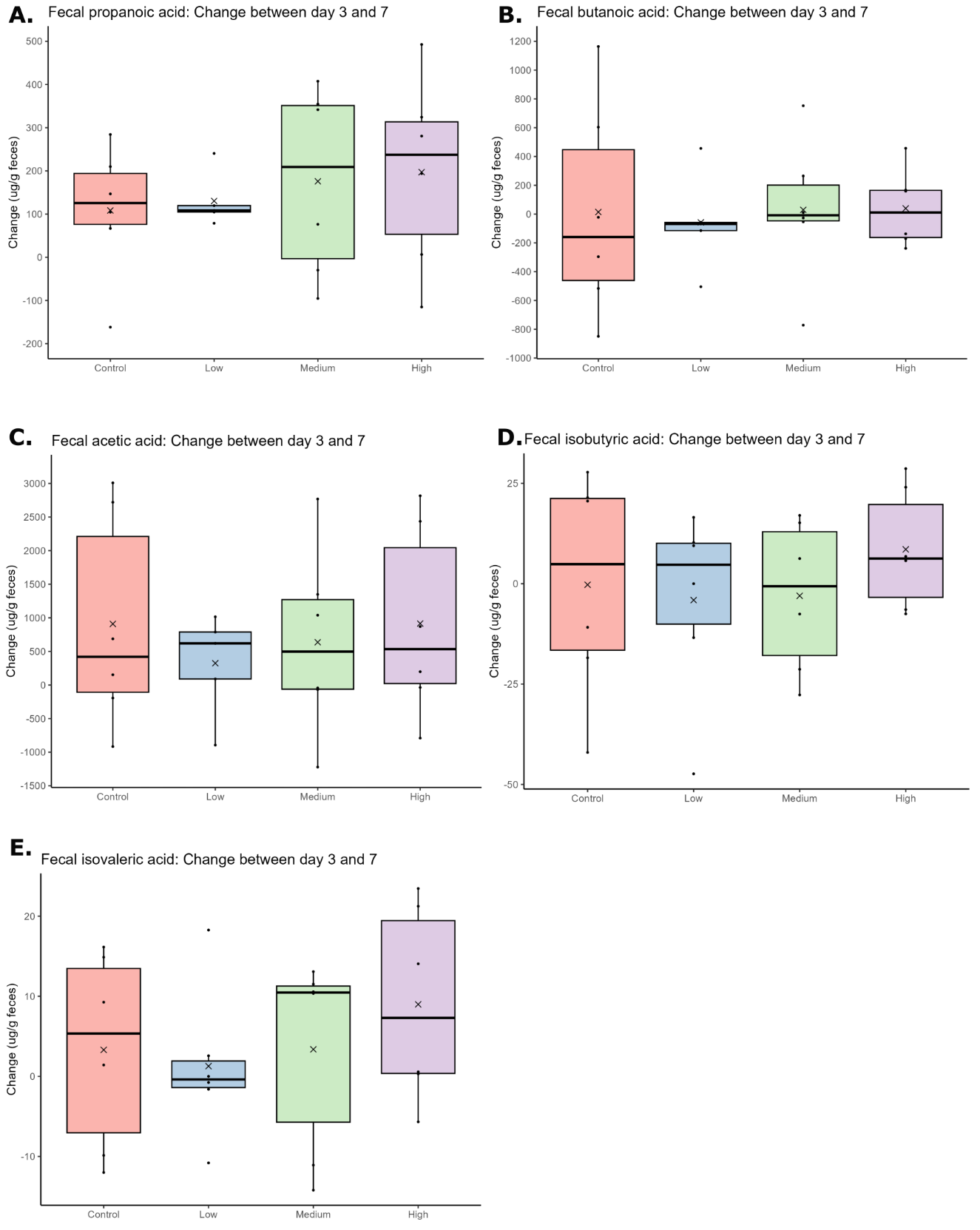
**

*Supplementary figure 8. Change in the fecal level of propionate (A), butyrate (B), acetate (C), isobutyrate (D), and isovalerate (E) between Day 3 and Day 7 for all groups. Differences between the groups were tested through two-way ANOVAs (non-significant). For isobutyrate and isovalerate, values below the detection limit were set to 0 before calculating the differences between the days.*


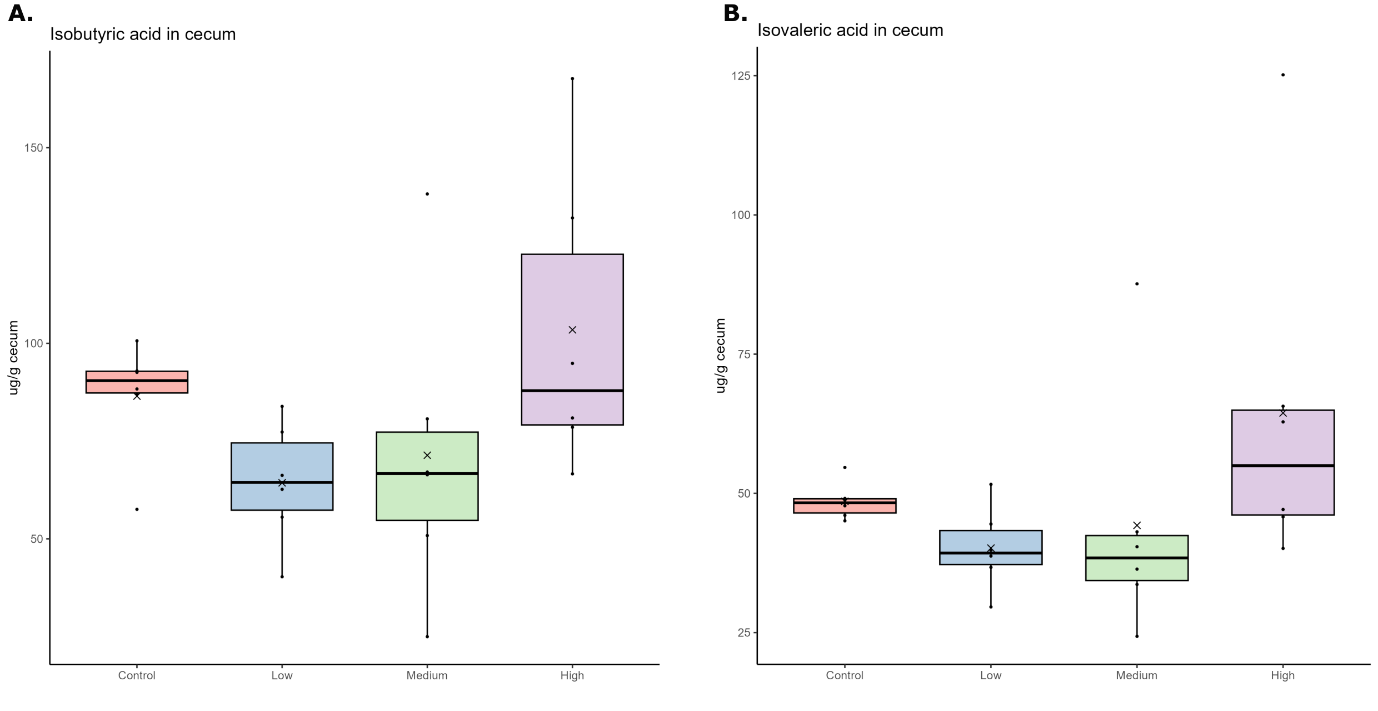


*Supplementary figure 9. Cecal levels of isobutyrate (A) and isovalerate (B) for all groups. Differences between the groups were tested through a one-way ANOVA for isobutyrate (non-significant) and a Kruskal-Wallis test followed by a Dunn’s test for multiple comparisons for isovalerate (non-significant after correction for multiple comparisons).*

*
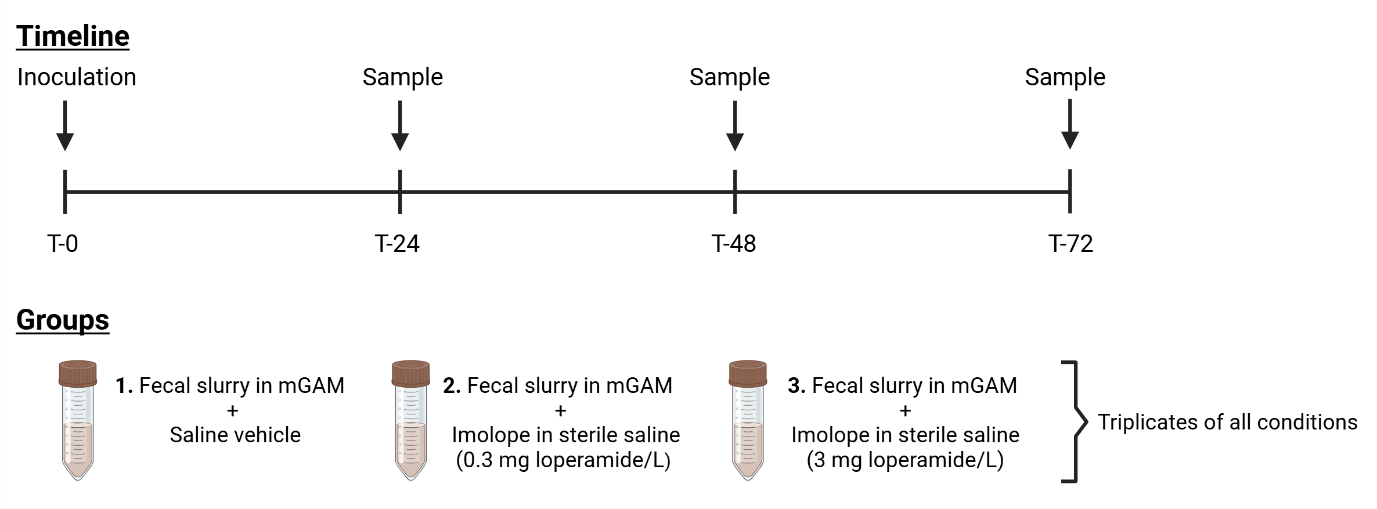
*

*Supplementary figure 10. Overview of the in vitro fermentation experimental design.*


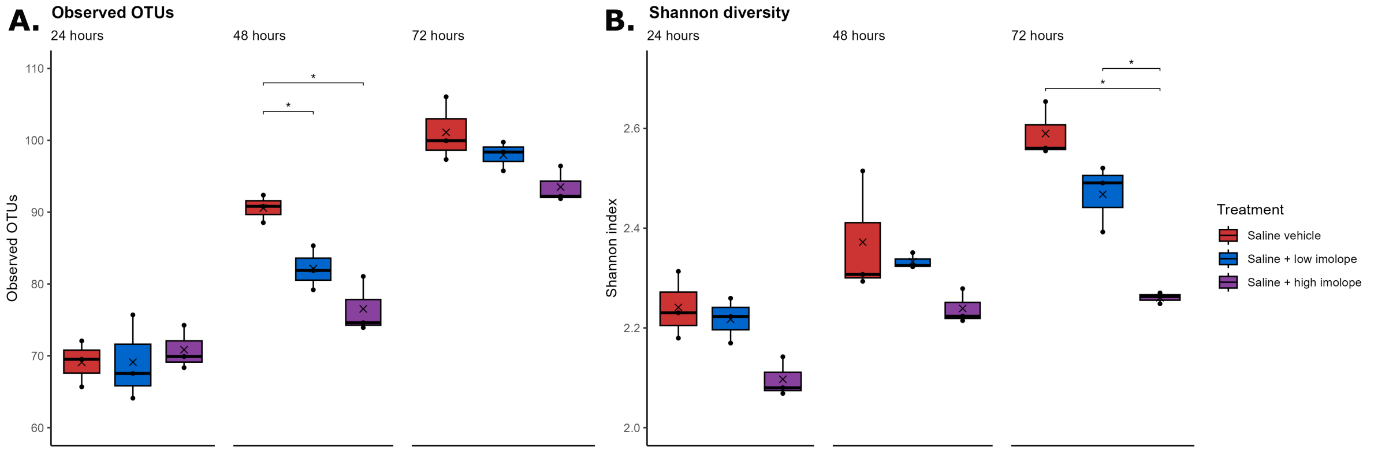


*Supplementary figure 11. The number of observed OTUs (A) and the Shannon index (B) in the different in vitro groups after 24, 48, and 72 hours of anaerobic fermentation. Differences were tested with a two-way ANOVA and unpaired t-tests for post hoc analysis.*

*
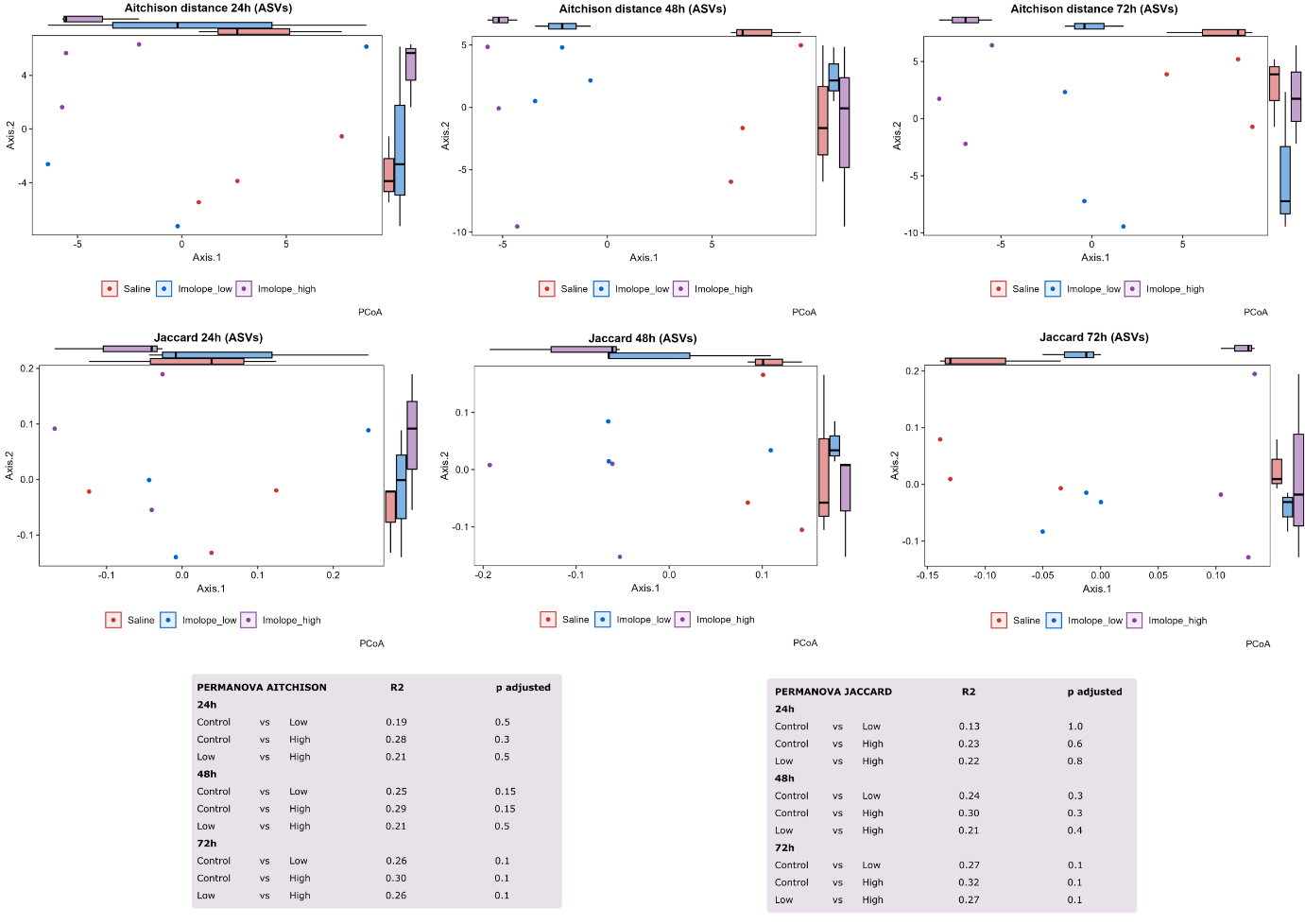
*

*Supplementary figure 12.* *PCOA plots illustrating the Aitchison distance (top panel) and Jaccard distance (bottom panel) between the microbiotas of the different in vitro conditions after 24, 48, and 72 hours of anaerobic fermentation. Dots represent individual samples, and marginal boxplots are included to illustrate the data distribution along the two axes. Differences between the groups were tested with pairwise PERMANOVAs with FDR adjustment for multiple comparisons. The R^2^ values indicate the proportion of variance explained by the model.*


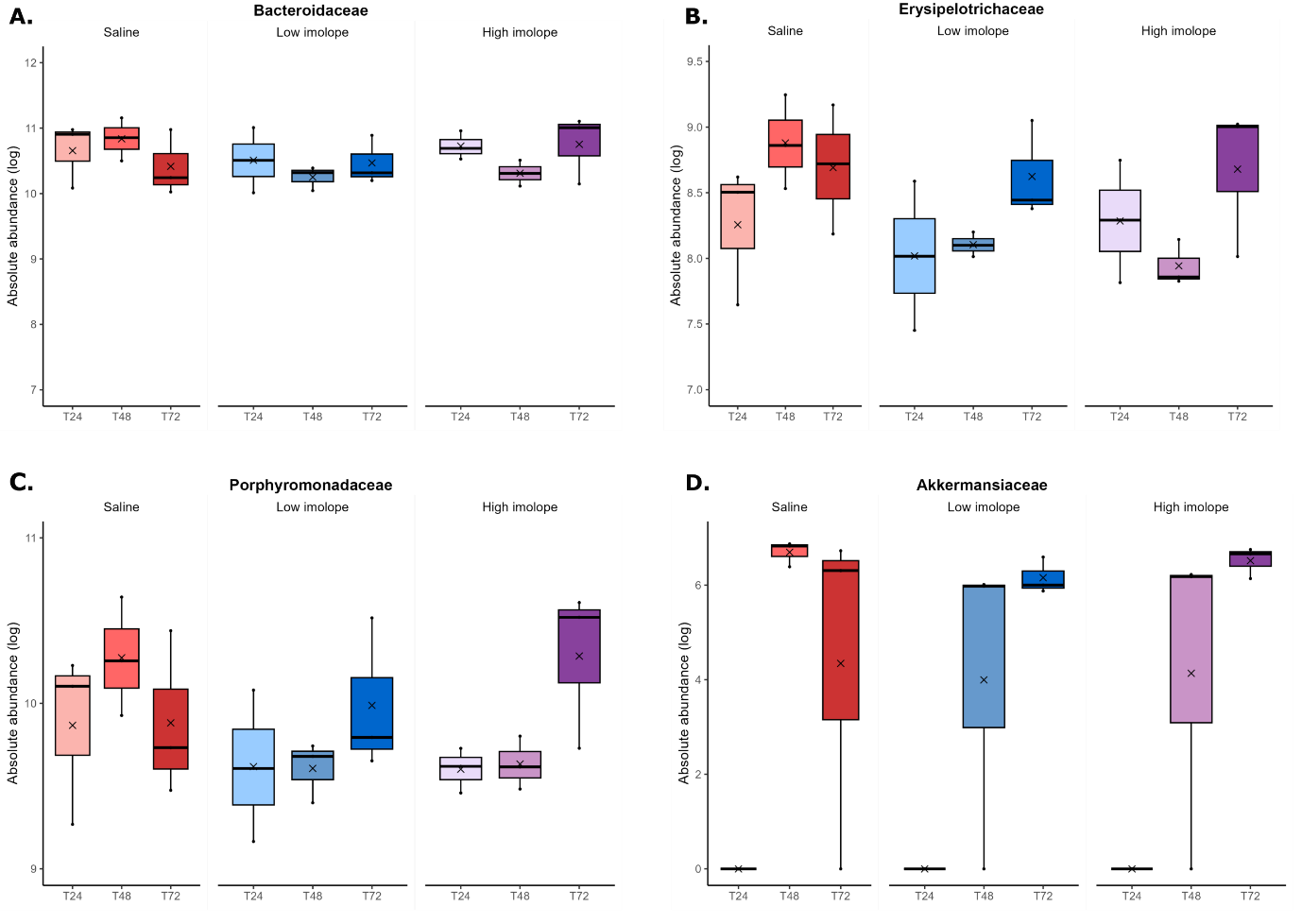


*Supplementary figure 13. The absolute abundance of Bacteroidaceae (A), Erysipelotrichaceae (B), Porphyromonadaceae (C), and Akkermansiaceae (D) after 24, 48, and 72 hours of anaerobic fermentation for all in vitro conditions. For Bacteriodaceae, Porphyromonadaceae, and Erysipelotrichaceae differences were tested through two-way ANOVAs, followed by paired t-test with FDR adjustment for multiple comparisons. For Akkermansiaceae, differences between the days were tested through a Friedman test, followed by paired Wilcoxon tests with FDR adjustment for multiple comparisons. Before LOG transforming absolute abundances, a psedudocount of 1 was added to all samples to handle any zeros before transformation.*
